# Supplementary material for: Mechanism of the noncatalytic oxidation of soot using in situ transmission electron microscopy
Source: Nat Commun. 2023 Oct 6;14:6256. doi: 10.1038/s41467-023-41726-4 (PMC10558545; doi:10.1038/s41467-023-41726-4)
Supplement: Supplementary file 1 — Supplementary Information [file 41467_2023_41726_MOESM1_ESM.pdf]

Supplementary information for

Mechanism of the noncatalytic oxidation of soot using in situ transmission electron microscopy

Ming Gao[a, c, d], *Yongjun Jang*[b], *Lu Ding*[a, d, *], *Yunfei Gao*[a, d], *Sheng Dai*[b, *], *Zhenghua Dai*[a, d], *Guangsuo Yu*[a, d], *Wenming Yang*[c, *], *Fuchen Wang*[a, d, *]

[a] *Institute of Clean Coal Technology, East China University of Science and Technology, Shanghai 200237, P.R. China*

[b] *Key Laboratory for Advanced Materials and Feringa Nobel Prize Scientist Joint Research Center, Institute of Fine Chemicals, School of Chemistry & Molecular Engineering, East China University of Science and Technology, Shanghai 200237, P.R. China*

[c] *Department of Mechanical Engineering, National University of Singapore, Singapore 117576, Singapore*

[d] *Engineering Research Center of Resource Utilization of Carbon-containing Waste with Low-carbon Emissions, Ministry of Education, Shanghai 200237, P.R. China*

---

[*] Corresponding Author.

E-mail address: dinglu@ecust.edu.cn (L. Ding), shengdai@ecust.edu.cn (S. Dai), mpeywm@nus.edu.sg (W. Yang), wfch@ecust.edu.cn (F. Wang).

Supplementary Figures

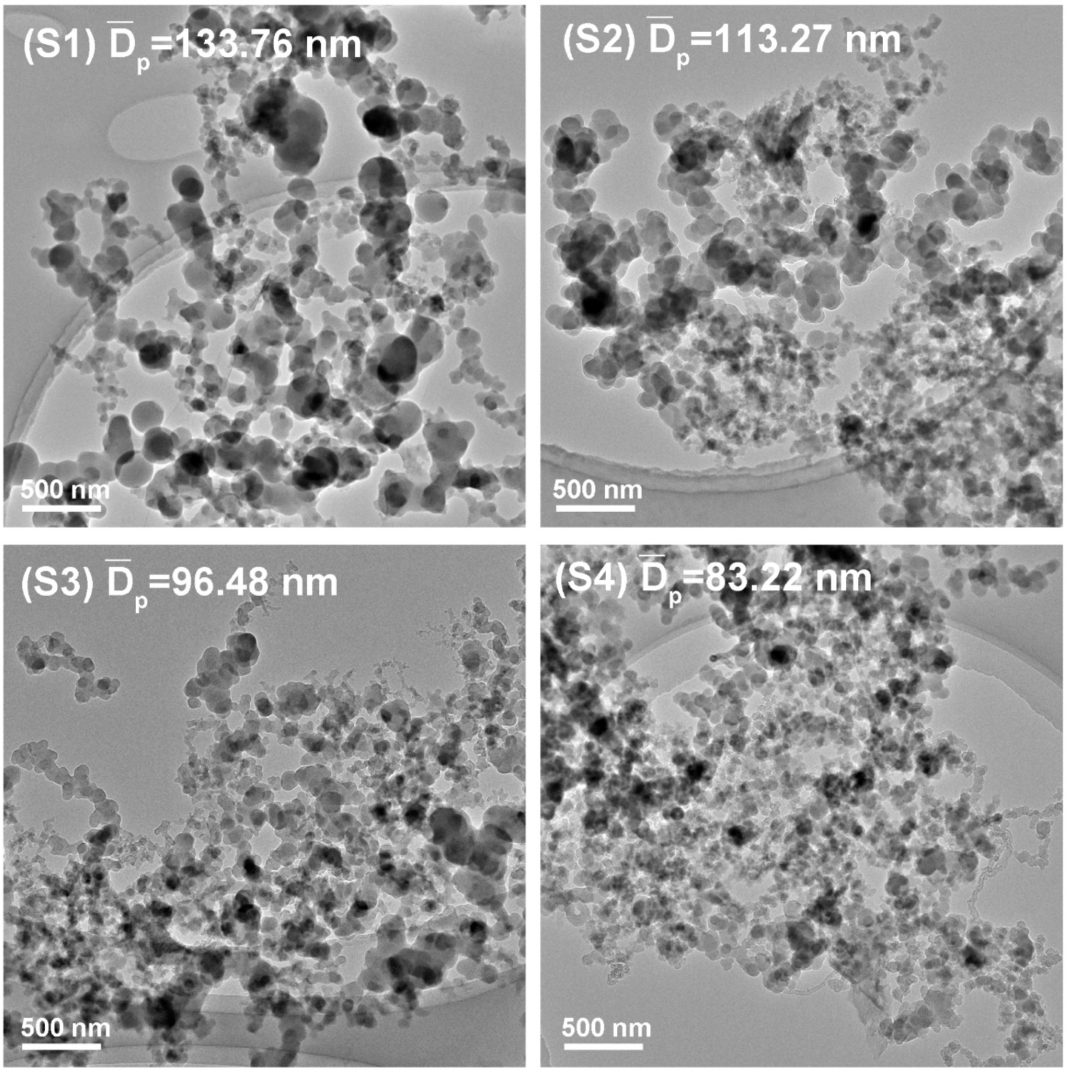

Fig. S1 Morphology of soot samples. All soot samples, (S1), (S2), (S3), and (S4) consist of particles of different maturity. The particle size decreased with the increasing of O_2/CH_4

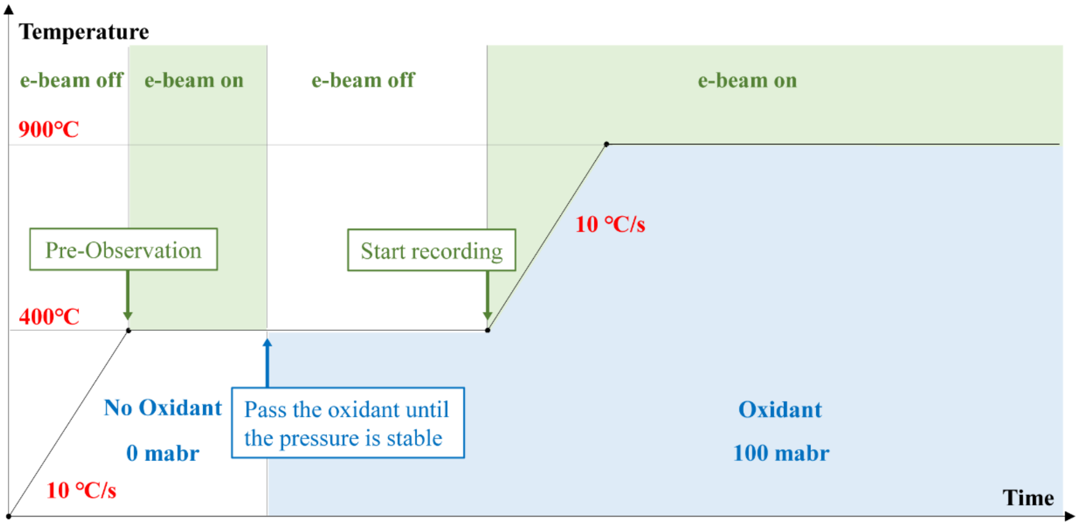

Fig. S2 In-situ oxidation process. Oxygen was pre-contacted with soot particles. The reaction was controlled by temperature.

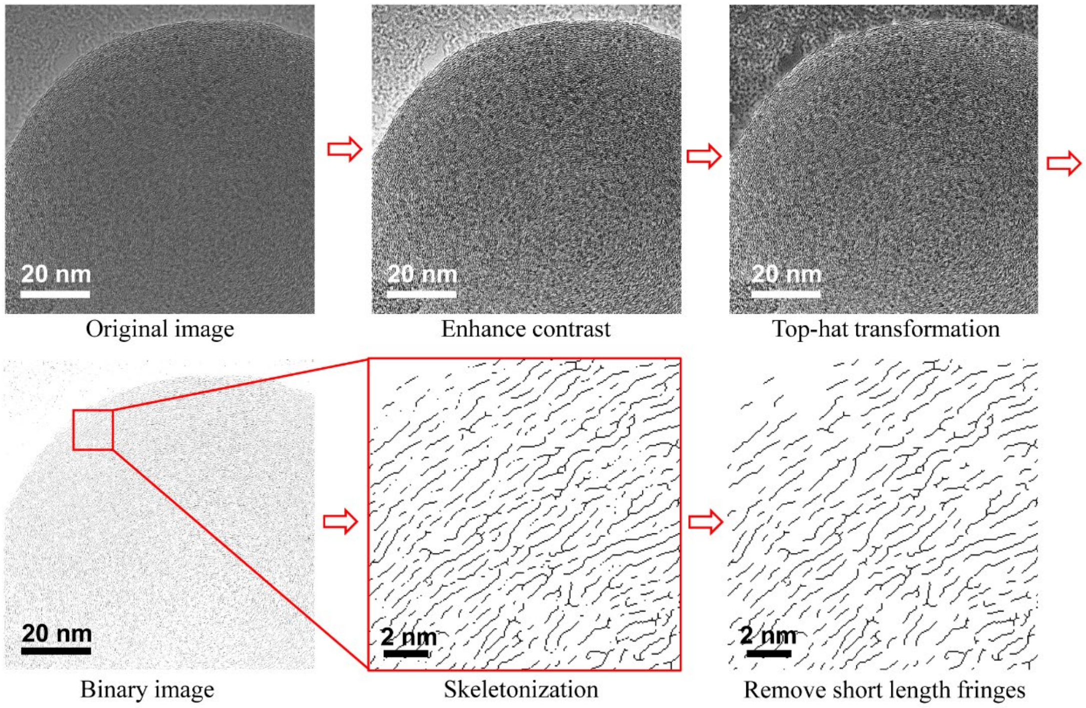

Fig. S3 Fringe analysis process. The original images were processed by contrast enhancement, top-hat transformation, binarization, and skeletonization

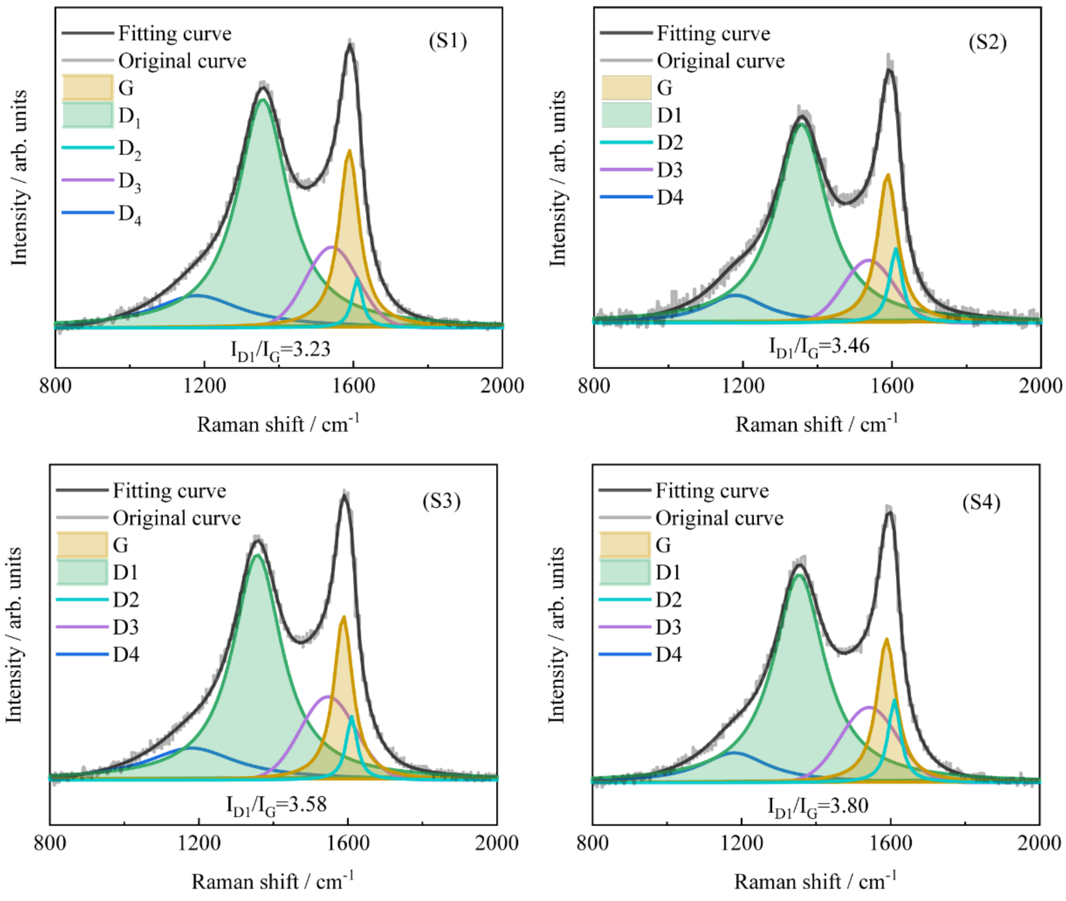

Fig. S4 Carbon structure of soot samples. The Raman results in the first-order Raman spectrum region of sample (S1), (S2), (S3), and (S4) were deconvoluted by five bands: G (1580 cm-1), D1 (1350 cm-1), D2 (1610 cm-1), D3 (1550 cm-1), and D4 (1180 cm-1). The integral band area ratio of the D band to G band (ID1/IG) was used to quantify the degree of graphitization of soot samples. Source data are provided as a Source Data file.

Supplementary Discussion

The specific derivation processes of application of oxidation models in combustion modelling:

Three oxidation models were established by in situ TEM. The models correlate the oxidation processes with the evolution of particle structures, and can be selected by the maturity parameter. To apply the models to real combustion environment, two

approaches are proposed.

(1) In the situation that soot samples can be obtained, the maturity parameter is directly calculated from the nanostructure of soot sample. Consequently, the corresponding oxidation models can be selected by the calculated maturity parameter. The relationship between conversion rate, x, and time, t, can be obtained by oxidation experiments of soot samples under the specific conditions, which is designed by researchers. Furthermore, the activation energy, E_a, and pre-exponential factors, A, of Eq. (1) can be calculated by using the oxidation models in this study. These kinetic parameters can be applied to the real combustion modelling. Soot particle is substituted as a series of species at each stage of oxidation. Soot oxidation process is regarded as a part of the combustion mechanism. The reaction rate can be obtained by Eq. (11). S_{soot} can be given by a certain value. The reaction of soot oxidation can be coupled with detailed mechanisms of various fuels. At this stage of computation, there is no need to further consider the influence of particle structure.

(2) In the situation that soot samples cannot be obtained, the maturity parameter is calculated from the detailed soot formation model. Detailed soot models include gas phase reactions and particle dynamics. To calculate particle behaviours, the particle size distribution, N, can be described by population balance equations (PBEs). PBE can be solved using the method of moments and coupled with fluid dynamics, for specific combustion calculation. N at certain time, t, can be described by discrete form of PBE as Eq. (S1) [1].

$$\frac{\partial N(\mathbf{y})}{\partial t} = R(\mathbf{y}) + G(\mathbf{y}) + W(\mathbf{y}) + F(\mathbf{y}) \quad \text{(S1)}$$

where $R(\mathbf{y})$, $G(\mathbf{y})$, $W(\mathbf{y})$, and $F(\mathbf{y})$, are the source terms of inception, coagulation, surface reaction, and fragmentation, and **y** is a series of the elements of soot type space, such as carbon atom numbers, volume, and surface area. Soot oxidation is contained in the source term of surface process. For example, the simplified reaction rate equation can be described as follows.

$$\frac{\mathrm{d}W\left(S_{\mathrm{soot}}\right)}{\mathrm{d}t}=r\left(\left(S_{\mathrm{soot}}+\delta S_{\mathrm{soot}}\right)N\left(S_{\mathrm{soot}}+\delta S_{\mathrm{soot}}\right)-S_{\mathrm{soot}}N\left(S_{\mathrm{soot}}\right)\right) \tag{S2}$$

where S_{soot} is the surface area of soot particles. The evolution of S_{soot} varies by oxidation models as shown in Eq. (16), (21), and (25). Reaction rate, r, can be expressed as a function of S_{soot} according to Eq. (11). Although the nanostructure of soot is not available, it is possible to calculate the carbon atom number and C/H ratio of the particle. The maturity parameter can be calculated by the proposed approach, and then the corresponding oxidation model can be selected through the maturity parameter.

Due to the typically small size of soot particles, their corresponding Stokes numbers are very small, which means the effect of soot on flow field is negligible. Therefore, under combustion conditions, soot particles are often described as no slip species, with their macroscopic velocity being the same as the flow field. However, the surface reactions of soot particles, especially oxidation, can impact the gas-phase components in the combustion environment. The oxidation models established in this study enables the correction of oxidation rates in chemical reactions kinetics, leading to a more accurate simulation of combustion.

Supplementary Methods

The calculation of the primary particle sizes of samples:

The boundary of each primary particle was fitted to an ellipse by a manual approach. The maximum Feret's diameter, d_{max} and the minimum Feret's diameter, d_{min} of the ellipse, were obtained by software ImageJ. The primary particle size, $\bar{D}_{\mathrm{p}}$, of the soot sample was the average of all the d_{max} and d_{min} of samples, as Eq. (S3).

$$\bar{D}_{\mathrm{p}} = \frac{\sum_{i=1}^{n} d_{i,\mathrm{max}} + \sum_{i=1}^{n} d_{i,\mathrm{min}}}{2n} \quad \text{(S3)}$$

Where i is the $\mathrm{i_{th}}$ primary particle, and n is the total number of primary particles measured. In this study, more than 2000 particles from 4 samples were measured.

References

1. Shaohua, W., *Numerical Study of Soot Formation and Oxidation Processes*. 2018, National University of Singapore (Singapore).
